# A Brief Sketch of the Life, Character, and Writings of William Charles Wells, M. D., F. R. S., &c., &c.

**Published:** 1850-01

**Authors:** Elisha Bartlett

**Affiliations:** Professor of the Theory and Practice of Medicine, in the University of Louisville, Ky.


Art. III.—*A Brief Sketch of the Life, Character, and Writings of William Charles Wells, M. D., F. R. S., &c., &c.* An Address delivered before the Louisville Medical Society, December 7th, 1849. By Elisha Bartlett, M. D., Professor of the Theory and Practice of Medicine, in the University of Louisville, Ky.

In the Introductory Address to the Medical class, which I had the pleasure of making, a few weeks ago, I took occasion to speak of the pleasures and the benefits which the practical physician might derive, from the cultivation of studies and tastes not immediately and exclusively connected with his own occupation. I expressed the opinion, that a reasonable devotion to these studies, and a reasonable indulgence in these tastes, would not only improve the character, and augment the sum of his happiness, by calling into exercise the finest and noblest powers of his mind ; but that he might, also, thereby be rendered a more successful and accomplished practitioner of his art ; that he might thus be made, not only a wiser and a better man, but a more skilful physician. Amongst the studies which I then particularly enumerated, and nearest allied of them all, to his practical duties, was the history and the literature of his own science and art ; and so much, especially, of this history and literature as are contained in the written lives of the most distinguished cultivators of medical science. I do not know any more agreeable study than that of biography ; and although the annals of our own science, when compared with those of politics, general literature, belles-letters, and the arts, cannot be said to be rich in this species of composition, they are still sufficiently so to furnish a fertile source of instruction and delight. I propose to occupy the time which you have assigned to me, on the present occasion, with a short history of the life and the labors, and with such a delineation and estimate of the character, as the study of this life and these labors will enable me to make, of an individual, less known, I am sure, than he deserves to be, especially to American physicians :—I mean William Charles Wells.

And I take up this subject, not as a piece of irksome drudgery, but as a labor of love. I had just finished reading, when I prepared this sketch, for the first time, the volume which contains the record of his life and the results of his labors; and my mind had been so interested and my feelings so warmed by its perusal, that the business before me became a pleasure, and not a task. I cannot doubt that I shall awaken in your bosoms emotions and sentiments corresponding in some degree to those which have been kindled in mine.

Dr. Wells was an American. He was born in Charleston, South Carolina, in May, 1757. His parents were both natives of Scotland. His father, who was a good general scholar, having been unsuccessful in some mercantile scheme, in Charleston, set up the business of a bookseller and bookbinder; he also printed a newspaper. He was a zealous and uncompromising tory; and he took great pains, and this with entire success, to educate his son William in the same political faith.

The son was sent to a grammar-school, in Dumfries, Scotland, before he was eleven years old, where he remained for nearly two years and a half. From Dumfries he was sent to Edinburgh, in the autumn of 1770, where he attended several of the lower classes of the University, and where he also learned drawing. He returned to his native town in 1771, and soon afterwards commenced the study of medicine, with Dr. Alexander Garden, the principal physician of Charleston; with whom he remained, a diligent student, for more than three years. The revolutionary contest between the American colonies and Great Britain had then fairly commenced; young Wells's father had already been obliged, on account of his political principles, to leave the country; and the son, himself, for similar reasons, soon after followed him. He arrived in England in the autumn of 1775; and, in the beginning of the following winter, commenced his regular medical studies at Edinburgh. In the autumn of 1778, he visited London, attended a course of Dr. William Hunter's lectures, and took instructions in practical anatomy. He was now appointed surgeon to a Scotch regiment in the service of Holland. He embarked for the Hague to take possession of his post early in 1779; but, soon after, becoming involved in some personal difficulties with the colonel of his regiment, he resigned his commission, and was discharged from the service. Immediately afterwards, in the beginning of 1780, he went to Leyden, where he spent three months, mostly occupied in the composition of his Thesis. In the autumn of the same year, he received at Edinburgh his degree of Doctor in Medicine.

In consequence of the deranged state of his father's concerns, in Charleston, growing out of the revolutionary war, he again visited this country, in the beginning of the year 1781. Charleston was at this time in the possession of the king's troops; but in December, 1782, in consequence of the evacuation of the garrison, Dr. Wells removed to St. Augustine, in East Florida, taking with him a printing press and types. He there commenced a weekly newspaper; and became, also, captain to a body of volunteers, raised for the purpose of resisting some threatened attacks of the Americans. The preliminaries of peace between the two countries having been signed, Dr. Wells returned to Charleston; and immediately on his arrival, was arrested on a private suit, originating in a transaction of his brother's, and committed to prison, where he remained upwards of three months. As soon as he succeeded in procuring his release, he again left for St. Augustine, and very narrowly escaped death by the wreck of his vessel, on the passage thither. In the summer of 1784, he returned to London, preparatory to his settlement in that city; where, after spending three months, in the spring of 1785, in Paris, in the words of his own sketch of his life, he had the name of Dr. Wells affixed upon the door of a lodging which he had hired. His father's property had now almost entirely disappeared, and he was obliged to borrow £130 from one of his friends, to enable him to commence his career, as a London physician. This career proved anything but prosperous. For several years he hardly took a single fee—and it was ten years, before his income from every source, including an annual gratuity of £50 from the Finsbury Dispensary, to which he had been appointed one of the physicians, reached the sum of £250 per annum; and during this period he had been obliged to increase his debt to about £600. In 1795, his professional receipts became equal to his moderate expenditures, and in the next five years he succeeded in paying off a small portion of his debt. From 1801 to 1812, his income fluctuated between £235 and £455; and, from regular fees alone it never reached £600. He finally paid the whole amount of his debt; and when he was obliged, by sickness, to relinquish his practice, he had, in his desk, for he never kept a banker, nor invested any money in the funds, about £350. This, with his books, his little plate and furniture, and a gold Rumford medal, made up the sum of his worldly fortune.

In 1800, he was seized with a slight fit of apoplexy, from the effects of which he never fully recovered.—About the year 1812, he began to suffer with symptoms of hydrothorax; and these continued, with some fluctuations in their degree of severity, but with gradually increasing intensity until the 18th of September, 1817, when he expired.

Such is a rapid enumeration of the leading events in the external life of Dr. Wells. There is very little about them either brilliant or imposing, and in order to understand at all the importance and the value of his life, we must turn to the study of his works.

In 1799, Dr. Wells addressed a letter to Lord Kenyon, in relation to certain alleged abuses existing in the official conduct of the Fellows of the Royal College of Physicians in London; and in the organization and by-laws of the college. This letter occupies nearly one hundred and forty pages of the edition of Dr. Wells's works, from which most of the materials for this sketch are derived; it is written with very great ability, and it exhibits in a clear and strong light, some of the most prominent elements in the character of Dr. Wells. For these reasons I intend to notice it, and some of the circumstances which called it forth somewhat fully. Apart from the connexion of these with this letter, and with the history of Dr. Wells, they can hardly fail, I think, to be of some interest to the American reader.

The Royal College of Physicians in London is of great antiquity, and it may certainly be regarded as one of the most distinguished associations of medical men that has ever existed. It was incorporated in 1518, during the reign of Henry viii, through the influence of the learned Linacre with Cardinal Wolsey. The Fellows of the College, beside being endowed with the usual privileges of close corporations, were empowered either by the terms of the original charter, or by successive additions to it, to examine into the qualifications of all physicians throughout England, except the graduates of Oxford and Cambridge; to search apothecaries' wares; to license lunatic houses; and in the exercise of their powers to commit offenders to any prison in London except the tower. No persons were allowed to practice medicine unless they were "*profound, sad, discreet, groundedly learned, and deeply studied in physic.*" The object of the founders of the college, and of the British legislature, in its incorporation, was to elevate and improve the character of the medical profession, and to protect the public, as far as possible, against the practices of ignorant and incompetent physicians. It is very evident, however, that even during the early periods of its existence, either through the selfishness of its Fellows, or from that easy sophistry, which so often convinces men in power, that their own elevation and supremacy are necessary to the interests and the welfare of those who are subject to them, the college overstepped the principal objects of its institution, and converted the powers, which had been bestowed upon it solely for the public good, into means and instruments for advancing the private and selfish ends of its Fellows. One of its earliest acts, tending directly towards the accomplishing of these ends, was that of limiting the number of its Fellows; thus excluding the great body, even of its own licentiates, from the honors and emoluments, directly or indirectly growing out of the Fellowship of the college. An arbitrary and invidious distinction, unauthorized by either the objects or the terms of the charter, was thus established between the Fellows and the licentiates of the college. Amongst other assumptions of authority, the college arrogated to itself the power of establishing conditions of admission, amongst both its Fellows and its licentiates, of the most unjustifiable and arbitrary nature. One of these was, that no person should be admitted either as a Fellow or a licentiate, who had ever been an apothecary, or sold drugs, or who had ever practiced midwifery as a part of his profession. These usurpations gave rise, as might have been expected, to complaints and resistance, on the part of those who felt themselves aggrieved by these outrages upon their feelings and their rights; and we accordingly find, that almost the entire period of the existence of the college has been marked by heart-burnings, jealousies, quarrels, and law suits, between the different classes of its members. Its Fellows were generally men of high professional rank, and extensive influence; some of them were almost always connected with the court; principles of justice, generosity, and right operated feebly upon the minds of men excited and influenced by strong and immediate personal interests; and it is not difficult to understand, why, in these contests, the college should have been the successful party. It appears, however, that notwithstanding these circumstances, the college did not always escape censure, even from the high courts, to which it appealed for the maintenance and protection of its assumed powers. In the year 1638, it was admonished by Chancellor Jeffries, on account of subverting the original intent of its charter.* The college was again censured in 1700, by Chancellor Somers; and in 1768, Lord Mansfield cautioned against narrowing its grounds of admission, as it seemed to have done, so that a Bœrhaave, if a resident in London, would have been excluded from its Fellowship.†

Amongst the distinguished persons who had quarrels and contests with the college, were Dr. Fothergill and Dr. William Hunter.

Although John Mason Good was a graduate of an English University, and aided Sir Henry Halford in the composition of his celebrated oration, he was refused admission to the full honors of the college, and placed among its *permissi*, because at one period of his life, he had been a general practitioner.

Catholics and dissenters were excluded from its Fellowship, the terms of admission requiring an assent to the church articles.

Sir Gilbert Blane constantly refused the proffered honor of a Fellowship.

---

*London Lancet, vol. xii, p. 108. †Ibid.

Without going any farther into the history of the college, I will now return to the letter of Dr. Wells, and to the circumstances which led him to write it. In the year 1797, he mentioned to two of his friends, both of them Fellows of the society—Dr. David Pitcairn, and Dr. Matthew Baillie—his intention of applying to the college for an examination of his fitness to become a Fellow. On the 29th of September, a motion was accordingly made by Dr. Pitcairn, and seconded by Dr. Baillie, that Dr. Wells should be admitted to this examination. The motion was resisted, and finally defeated—there being only ten votes out of twenty-three in its favor. In the following year, Dr. Wells, with the assistanee of his two friends, made another effort to obtain an examination, and was again defeated. This harsh treatment on the part of the college seems to have been the immediate occasion of Dr. Wells's letter. After entering somewhat fully into the history and merits of the case of Dr. Stanger, who had then recently been unsuccessful in an application for a like examination, Dr. Wells takes up the subject of his own grievances. As to his qualifications for a Fellowship, so far as knowledge was concerned, he says:—"Nine years previously to my being proposed by Dr. Pitcairn, I had undergone the trials of fitness, to which licentiates are subjected before admission to practice, and if I may venture to credit what was said by Sir George Baker, and the censors who examined me, I had passed through those trials with more than ordinary ease. In the interval, I had become a member of the Royal Society, the certificate of my fitness for which was signed by the late and present residents of the college, Sir George Baker, and Dr. Gisborne, and by four others of the present Fellows of that body. During the same interval I had endeavored to extend the boundaries of our knowledge in various parts of natural philosophy; and two of my attempts of this kind, certainly not the most considerable, had been recorded in the printed transactions of the Royal Society. As I had thus demonstrated industry at least, in the cultivation of sciences collateral to medicine, it is not probable that I had been inattentive to the study of my own profession, since my peace of mind necessarily depended upon my understanding it. Nor had my opportunities of gaining experience in it been very small; for I had been eight years a physician to an extensive establishment for the relief of the sick poor, and I had also been physician, for some time, to another institution of the same kind, but still more considerable. From all these circumstances, I think it will readily be allowed by your Lordship, that it was not likely I had become less learned since passing the trials of a licentiate."*

In connexion with his moral fitness for the place which he desired, he thus speaks of the persons who made and seconded the proposal for his examination:—" One of these gentlemen must already be well known to your Lordship. I cannot, however, refrain from saying respecting him, that the son of the gallant Major John Pitcairn, who died the glorious and enviable death of a soldier fighting for his country, and the adopted son of the high-minded, upright, and generous Dr. William Pitcairn, must have every title to the strictest honor, which inheritance, education, and domestic example can bestow. But why do I speak of titles, after his countrymen had acknowledged his complete possession of that most invaluable property, and had in consequence, as well as from their high opinion of his learning and skill, placed him at the head of the profession of medicine, in the metropolis of Great Britain." Dr. Wells adds, in a note, this commentary:—" Two circumstances must concur to place a Physician at the head of his profession in London:—1.

*Works of Dr. Wells, p. 330.

Great employment, which alone is certainly not sufficient for that purpose, as it is often possessed by persons of no considerable ability. 2. Respect from other Physicians, indicated by their frequently requesting his aid in their practice. This can arise only from a high opinion of his honor and skill, of which qualities in a Physician, scarcely any but those of his own profession have either opportunities or capacity to judge rightly. Dr. Pitcairn, from the death of Dr. Warren to his own unfortunate illness, was indisputably the Physician in London, in whom these circumstances existed together in the greatest degree." "He who seconded the proposal," says Dr. Wells, "Dr. Matthew Baillie is more upon a level with myself, in regard both to age and length of residence in London. Somewhat therefore of the obscurity which involves almost every young Physician may have hitherto concealed him from your Lordship's notice. But that obscurity is fast dissipating, and he must soon, my Lord, very soon, appear to your view, with all the just proportions and accurate lineaments of a man of integrity, learning, and great professional skill."* This judgment was pronounced just at the period when the sun of Dr. Baillie's reputation was rising to its zenith; and one can hardly fail of being struck with the broad and touching contrast presented in the lives—from this time onwards—of these two men. Dr. Baillie may fairly enough be taken as the fittest type and representative of one of the noblest classes of men, that have ever blest and adorned humanity—the leading practitioners of medicine in Great Britain, during the last century. Skilled alike in ancient and in modern lore—grave and polished in their manners—courteous in their intercourse with all classes, but loyal to the stern and tyrannous social aristocracy of their land—upright,

*Works of Dr. Wells, p. 324.

benevolent, generous, industrious, skillful, and humane—worshippers of God, and lovers of men—they filled up faithfully the measure of their laborious and beneficent lives, and left to their survivors memories embalmed in the fragrant amber of gratitude and love. Dr. Baillie's father was a professor in the University of Glasgow—and his mother was a sister of the Hunters; Miss Joanna Baillie was his sister, and he married the daughter of the celebrated Dr. Denman. He studied his profession under the immediate personal direction of his uncle, William Hunter, and at the early age of twenty-two years, in connexion with Mr. Cruikshank, he continued the anatomical lectures at Mr. Hunter's school in Windmill street. Dr. Pitcairn, then at the head of the profession in London, made his friend, Dr. Baillie, the heir of his immense and lucrative practice; and under such auspices, and with his high qualifications, and his many excellent qualities of head and of heart—his thorough knowledge of his profession—his untiring industry—his exclusive devotion to his business—his clear, strong sense—his sound judgment—his simple, unostentatious, and dignified manners—his truthfulness—his generosity—his kindness to those less favored by fortune than himself—it is not strange that he rose so rapidly to opulence and fame. He died in 1823, having lived for twenty years in the full blaze of professional popularity; long a favorite attendant in the household of his sovereign, and his services in constant requisition by the noble, the fashionable, and the great. During a considerable portion of these same years of prosperity and success, Dr. Wells was also a London practitioner;—with high qualifications—a favorite friend both of Baillie and Pitcairn—but how widely different were the circumstances which attended his career, and the fortune which awaited him! *with five friends*, as he so sadly and touchingly says, *in the world;* abstemious in his habits, and almost sordid, to use his own words, in his manner of living—struggling on from dismal year to year, in this boiling and turbid ocean of London life, with shattered and failing health, and scanty resources, but still paying scrupulously his income and property tax, and allowing for a good many years an annuity of £20 to a female relation; after a night passed in the open air of his garden at Surrey, plodding his way on foot to the houses of his few patients, or to the hospital and dispensary—a solitary wanderer through the crowded thoroughfares of the metropolis; receiving, at least in one conspicuous instance, and in a matter closely connected with his highest aspirations, and near to his heart, only harshness, contumely, and neglect, where he deserved and should have found only kindness, respect, and honor; and all this dull and hard environment of his life uncheered and unilluminated by the light and the warmth of the domestic affections, and of home!

I have already alluded to Dr. Wells's loyal adherence to the cause of Great Britain in her war with the American colonies. It seems that amongst the objections made by some of the Fellows of the College of Physicians, against the admission of licentiates, it was alleged that the latter had become imbued with revolutionary and republican principles. It may easily be supposed that Dr. Wells would find no difficulty in clearing his own character from a charge of this nature. "Leaving, however," he says, "to more able advocates, what further defence may be deemed proper for the other licentiates, who have been charged with disloyalty by the members of the College, I shall now confine myself to a special vindication of my own character from so atrocious a calumny. If, my Lord, I speak with warmth upon this subject, I trust that I shall find an excuse in the energy of your own feelings. He that is wealthy may be robbed, without knowing that he has experienced an injury. But the poor man's all is often included in a single object, which, although to other eyes worthless and contemptible, may be to him the sole spring of joy and hope. Any attack upon it excites his utmost powers of resistance; its loss leaves him without bond to the world, or interest in its concerns. When we read of a rich man's despoiling a poor neighbour of his only property, 'one little ewe-lamb, which lay in his bosom, and was unto him as a daughter,' our sympathy with the sufferer is nearly as great, as if he had been a monarch unjustly expelled from his dominions. I may well then be allowed to feel acutely the attempt which has been made to strip me of almost my only possession, to which my title is founded upon paternal discipline and personal suffering, and has been illustrated by the whole tenor of my life."* He then narrates the circumstances of his conduct in Charleston during the war of the revolution. In a note to his letter to Lord Kenyon, he speaks with much feeling of the kindness shown to him, every day during his three months of imprisonment, by Mr. John Harleston, and his wife Mrs. Elizabeth Harleston, persons of rank and fortune in that country. A mob once surrounded Mr. Harleston's house in the night, threatening to destroy it on account of his protection of Dr. Wells. Mr. Harleston was from home; but his wife, with the spirit and dignity of a Roman matron, went out to the rioters, and told them, that her husband and herself had done nothing towards Dr. Wells but their duty, and that they should not be prevented from continuing to perform it, by any menace whatever. "One of those persons," adds Dr. Wells, "is since dead; the other still exists, an ornament to her sex. Excellent woman! enjoying in affluence, in the midst of thy children, and their children, the calm evening of a well spent life, and looking forward with a firm hope, inspired by our holy

---

*Works of Dr. Wells, p. 340.

religion, to another and a better state, though thou seemest already to possess as much of happiness, as is compatible with the infirmity of our present natures; it may yet afford thee some momentary satisfaction to know, that neither distance of place, nor intervention of time, hath lessened my sense of thine unspeakable goodness; and that at this moment my bosom heaves, and my eyes drop tears, while I reflect, that without thy tender cares concerning me, when sick and in prison, and far removed from those, whose duty it was to render me service under such distress, I might long ago have been numbered with the dead."*

Dr. Wells, near the close of his letter, enumerates several of the influences which act unfavorably upon the character of physicians, in the course of which he gives the following noble portraiture of Dr. Heberden. "Many of our physicians," he says, "have no doubt received little injury from the causes of the corruption of character, to which they have been exposed; and some few may have escaped their influence altogether. One of these few, Dr. William Heberden, I must conclude to have been well known to your Lordship, from the eulogy which you pronounced upon him, during the trial of Dr. Stanger's cause. He was probably, indeed, the only physician with whom you were intimately acquainted, and hence, from the natural error of attributing to a whole species the properties of its only individual we have seen, you might imagine that he possessed his many virtues in common with the rest of his class. But Dr. Heberden, my Lord, stands in a manner, alone in his profession. No other person, I believe, either in this or any other country, has ever exercised the art of medicine with the same dignity, or has contributed so much to raise it

---

*Works of Dr. Wells, p. 347.

in the estimation of mankind. A contemplation of his excellencies therefore can afford little help towards obtaining a just notion of the general worth of physicians. In speaking of a molehill, we would not employ terms that had relation to the immensity of a mountain.

"Were I, my Lord, possessed of talents adequate to the undertaking, I should here endeavor to describe at full length the character of that illustrious man. In this attempt, I should first mark his various and extensive learning, his modesty in the use of it, and his philosophical distrust of human opinions in science, however sanctioned by time, or the authority of great names. I should then exhibit him in the exercise of his profession, without envy or jealousy; too proud to court employment, yet undervaluing his services after they were performed; unwearied, even when a veteran in his art, in ascertaining the minutest circumstances of the sick, who placed themselves under his care, taking nothing in their situation for granted, that might be learned by inquiry, and trusting nothing of importance that concerned them to his memory. To demonstrate his greatness of mind, I should next mention his repeatedly declining to accept those offices of honor and profit at the British court, which are regarded by other physicians as objects of their highest ambition, and are therefore sought by them with the utmost assiduity. I should afterwards take notice of his simple yet dignified manners, his piety to God, his love for his country, and his exemplary discharge of the duties of all the private relations in which he stood to society; and I should conclude by observing that his whole life had been regulated by the most exquisite prudence, by means of which his other virtues were rendered more conspicuous and useful, and whatever failings he might as a human being possess, were either shaded or altogether concealed. After my description was finished, I should think it proper to say, that I had never been acquainted with Dr.

Heberden, and consequently could neither be dazzled by the splendor of his virtues, from approaching them too nearly, nor influenced in my opinion concerning them, by benefits he had already conferred upon me; and that standing, as he does, upon the verge of this state of existence, ready to wing his flight to another of glory, his ear must now be closed to the voice of flattery, had he ever listened to that siren, or were I base enough to solicit her aid, in the foolish expectation of receiving from him some future reward."*

In the memoir of his life, Dr. Wells says: "About four years ago Dr. Baillie asked me, in the name of the President of the Royal College of Physicians of London, if I had any desire to become a Fellow of it; to which I answered that I had none."†

Dr. Wells published an essay upon single vision with two eyes, and observations on several subjects in optics—rather speculative than experimental in their character, and leading to no very important or positive results. He furnished several short biographical sketches for the Gentleman's Magazine; several of his papers were published in the Philosophical Transactions, and in Thomson's Annals; and there are twelve papers on medical subjects, furnished by him, in the second and third volumes of the Transactions of a Society for the Promotion of Medical and Chirurgical Knowledge. The first paper, he says, which he ever wrote for the public, was an account of Mr. Henry Laurens, some time president of the American Congress, and which was published in a newspaper in 1780. He also furnished for the journals many political articles during the years 1780 and 1781; the most important of which was a paper upon the subject of military punishments—a paper which, he intimates, may have

*Works of Dr. Wells, p. 375. †Works of Dr. Wells, p. 40.

influenced General Balfour and Lord Moria in putting to death a Colonel Haynes, the propriety of whose fate was afterwards a subject of debate in the British House of Commons.* The memoir of his own life was dictated to his friend Mr. Patrick at intervals during his illness, after he had lost all hope of recovery, and while he was uncertain whether he should live to finish it, and when he was too feeble to speak long, or to write much.† He contemplated the publication of a metaphysical treatise; and he intended to have presented to the Royal Society several papers on Vision.

The work of Dr. Wells which has given him his chief scientific celebrity—which has written his name in the fair annals of science, beyond the reach of accident or time—is his *Essay on Dew.* A late brilliant writer quotes the concluding lines of Walter Savage Landor:

> "Pleased it remembers its august abodes,
> And murmurs as the ocean murmurs there,"

and then makes the following remarks:—"These are the far famed lines on a shell, which Wordsworth has imitated, and every body praised, and which, if they will not immortalize the name of Landor, nor embalm the poem of Gebir in which they occur, have assuredly immortalized and embalmed themselves. And never in remotest time, shall any one who has once heard or read them, gaze into the white depths of the child of ocean, or apply to his ear its polished coolness, and hear or seem to hear the faint and far-off murmur of the main, without imagining that these are the words which the gentle oracle is uttering, and this the meaning of the spiritual and mysterious music. They are among those rare lines which, giving to a common thought or belief an expression poetic and ideally perfect, stamp themselves at once

*Works of Dr. Wells, p. 43. †Works of Dr. Wells, p. 42.

on the heart and memory of the world. *Enviable the powers which by one true and strong line render oblivion impossible.* Who does not bless the nameless warrior who has left the noble epitaph: *Siste viator, heroem calcas*, as his sole memorial? So cheaply, sometimes, does genius purchase immortal fame!" What huge cart-loads of poetry—so called—we may continue in a similar strain, are yearly trundled into market; what countless acres of the same article—all the lines regularly marshalled—armed and equipped according to the laws of Parnassus—trotting off on their due number of feet—their heads bristling with capitals, and their tails jingling with rhyme—are every month spread out before us on the innocent pages of newspapers and reviews—to be read before breakfast, and forgotten before dinner; while one brief hour of happy inspiration has given to the short and simple lines on the burial of Sir John Moore an immortality as assured and irrevocable as that which awaits on the Paradise Lost and Macbeth. And analogous, at least, to these instances, is the character, and the history of the *Essay on Dew.* Certainly, it would be extravagant to place the Essay of Dr. Wells in the same rank and scale with the *Novum Organum* and the *Principia*, or to compare his discoveries with those of Galileo, and Harvey, and Jenner—but amongst the works which come next to these transcendant achievements of the human intellect, there is no one which has attained, as there is no one which deserves a more eminent position.

What a beautiful phenomenon is that of Dew! As soon as the diminishing rays of the declining sun allow the surface of the earth to lose something of its noon-tide heat, this silent distillation from the great alembic of the atmosphere begins; and through the evening and the morning twilight, and the serene watches of the night, every leaf of the forest, every blade of grass, and every flower of the field, gathers its beaded and transparent gems, to glitter like flashing diamonds, and to be exhaled like auroral incense in the rays of the early sun. And as if to give to this phenomenon an especial and particular beauty, it is witnessed only under cloudless heavens and in still nights; when the winds are hushed, and the stars are shining in the sky. What a delicious element would be lost from the manifold charm and glory of a summer dawn, if there was no dew on the grass and the flowers! And how would the breath of Aurora be robbed of its fragrance, and her roses of their freshness and their bloom!

Up to the time of Dr. Wells's researches, the true character of this process, the real causes of this phenomenon were entirely unknown. From the age of Aristotle, it had been a common observation, that dew appears in considerable quantities only on still and clear nights; and in the latter part of the last century, it was noticed by Dr. Wells, by Mr. Patrick Wilson, of Glasgow, and by Mr. Six, of Canterbury, that the formation of dew and of hoar frost is attended by the production of cold. Mr. Wilson and Dr. Wells, at this time, supposed that the cold was occasioned by the formation of the dew—that the latter was in some way the cause of the former. Musschenbrock and Dufay had studied the subject of dew, but had failed altogether to discover its true philosophy. Dufay and some others believed the deposition of dew to be an electrical phenomenon. Aristotle, Professor Leslie, of Edinburgh, and others, believed dew to be a species of rain, "formed in the lower atmosphere, in consequence of its moisture being condensed by the cold of the night into minute drops."* Gersten, who published an Essay on Dew in 1733, Noah Webster, of Connec-

---

*Works of Dr. Wells, p. 177.

ticut, and some others, adopted the opinion that dew consists in vapor emitted by the earth at night, and condensed by the cold.* Such were the prevailing doctrines upon this subject, when Dr. Wells commenced his investigations, under circumstances and conditions, that give a touching and melancholy interest to the scene. This was in the autumn of 1812. Dr. Wells was then fifty-five years old—a lonely, austere, and abstemious man—the distressing disease which in a few years terminated his hard and chequered career, already far advanced; his ankles swollen at the close of the day—on the slightest exertion panting with breathlessness and palpitation, and

---

*"As dew appears to collect only during fine clear nights, when the heavens glow with sparkling constellations, the ancients, in the infancy of science, imagined it to be actually shed from the stars, and therefore to partake of a pure and celestial essence. Hence the vulgar notion that *dew falls*, which has prevailed through all ages, and continues to tincture every language. * * The dew of heaven has always been regarded as a fluid of the purest and most translucid nature. Hence it was celebrated for that abstergent property which, according to the vulgar persuasion, enables it to remove all spots and stains, and to impart to the skin the bloom and freshness of virgin beauty. Like the elixir of later times, it was conceived to possess the power of extending the duration of human life; and Ammianus Marcellinus ascribes the longevity and robust health of mountaineers, in comparison with the inhabitants of the plains, chiefly to the frequent aspersion of dew on their gelid bodies. Dew was also employed as a most powerful agent, in all their operations, by the alchemists, some of whom pretended that it possessed such a subtile and penetrating efficacy as to be capable of dissolving gold itself. Following out the same idea, the people of remote antiquity fancied that the external application of dew had some virtue in correcting any disposition to corpulence. The ladies of those days, anxious to preserve their fine forms, procured this celestial wash, by exposing clothes or fleeces of wool to the humefaction of the night."

"The opinion that dew falls from the sky maintained its credit during the course of the middle ages. The alchemists even carried this idea so far as to fancy that, since the dew gradually vanishes in the progress of the day under the action of the solar rays, it then merely seeks by sympathy to regain its native seat in the highest heavens. Nay, some of those ingenious enthusiasts have not scrupled, in confirmation of their wild hypothesis, broadly to assert that a few drops of morning dew, being enclosed in an empty egg-shell, which is placed at the foot of a ladder resting against the roof of a house, the shell will become buoyant while the sun shines, and will mount along the ladder till it reaches the very top.

*Encyclopædia Brittanica. Article, Dew.*

his nights disturbed with restlessness and pain. Provided, as the principal parts of his apparatus, with some small thermometers, some plates of different kinds of metal, a few watch-glasses, and small pledgets of swan's down and wool, he repaired to the theater of his labors, which he describes in the following words. "Previously to mentioning the results of any of my experiments with these parcels of wool, I think it right to describe the place, where by far the greater part of my observations on dew were made. This was a garden in Surrey, distant, by the public road, about three miles from the bridge over the Thames at Blackfriars, but not more than a mile and a quarter from a densely built part of the suburbs on the south side of that river. The form of the garden was oblong, its extent nearly half an acre, and its surface level. At one end was a dwelling house of moderate size, at the other a range of low buildings; on one side a row of high trees, on the other a low fence, dividing it from another garden. * * Within it were some fruit trees, but, as it had not been long made, their size was small. Towards one end there was a grass-plat, in length sixty-two feet, and nearly sixteen broad, the herbage of which was kept short by frequent mowing. The rest of the garden was employed for the production of culinary vegetables."*

With the simple apparatus to which I have alluded, on the green sward of this little garden in Surrey, with only the silent stars and the blue sky above him, did the philosopher meekly and reverently enter the great temple of Nature—an earnest seeker of her secrets—an humble worshipper at her shrine. And the true offering which he laid upon her altar was blessed and accepted ; the veil which had hidden one of her most beautiful mysteries

*Works of Dr. Wells, p. 137.

was withdrawn; a new and one of the fairest pages in her great volume was opened at his invocation to the wonder and admiration of the world. Few and simple as were the means with which Dr. Wells conducted his researches, his experiments were so various, so direct, and so conclusive—so sagaciously devised and so admirably executed—that the whole philosophy and economy of the subject, which he studied, were completely and definitively settled. It would be difficult, I think, to find any other instance in the history of physical science, where a subject, hitherto so little understood, was so thoroughly and so satisfactorily investigated, by a single, unaided inquirer; and where so little was left, either for amendment or addition by subsequent observers. From the commencement of Dr. Wells's researches, in the fall of 1812, he continued his experiments, often through the whole night, returning to his professional labors in London during the day, frequently interrupted by his increasing ill-health, till near the close of the following year, when he became so infirm and feeble as to be obliged to discontinue altogether his visits to his garden.

"In the beginning of 1814, a considerable snow having fallen, I could not resist," he says, "the temptation of going for several evenings to Lincoln's-Inn-Fields, during a very severe frost, in order to repeat and extend some of Mr. Wilson's experiments upon snow." But he was soon obliged to desist, and his symptoms became so alarming, that his friend, Dr. Lister, earnestly urged him to remain quiet, and announced to him that, in all probability, a few months would terminate his earthly career.

"Upon receiving this opinion," he says, "I set about immediately composing my Essay on Dew, as my papers containing the facts on which my theory was founded, would, after my death, be altogether unintelligible to any person who should look into them. I labored, in consequence, for several months with the greatest eagerness and assiduity, fancying that every page I wrote was something gained from oblivion." And something it certainly was, gained from oblivion, and worth gaining from oblivion. This was his labor of love, and worthily did he accomplish it. He lived not only to complete his essay, but to publish a second edition. A generation has since passed away;—Science, with her manifold faculties—restless, unappeasable, longing for absolute and boundless dominion,—her forehead flushed with daily triumphs, and radiant with undying youth,—has extended in every direction the area of her empire, crowding and adorning it with her trophies;—art and industry have changed not only the whole face of nature, but the most intimate and vital relations of life and society, the intercourse of man with man, and the interchange and transmission of thought; but this handiwork of Dr. Wells still stands as he left it—not like the colossal calculus of Newton, holding in its stupendous embrace, alike the light dust on the balance, and the infinite universe of worlds; but, nevertheless, finished, faultless, and entire,—compact and perfect in itself,—graceful and imperishable as one of the granite obelisks of the Nile, resting its basis on the solid earth, and lifting its apex high towards the heavens. Such is the clear and concurrent verdict of scientific men; and as long as the earth in her annual circuit round the sun, proclaims, in the music of the spheres, the name of Galileo;—as long as the glory of Newton is set with the rainbow in the firmament;—as long as the fame of Harvey is spoken by every throb of the beating heart; as long as the lightning flashes forth from horizon to horizon the great discovery of Franklin; so long shall the hoarfrost and the dew, as through winter and summer, in each still and starry night, they gather and sparkle, over all the broad surface of the earth, upon hedgerows and fences, upon mountain and valley, upon field and forest and meadow, upon cottage-roof and temple-dome, keep green and unfading the name and the memory of William Charles Wells.*

---

* A critique on the essay appeared in the Quarterly Review for October, 1814, written, as it appears from a reply of Dr. Wells, by the learned and philosophical Dr. Thomas Young. The notice is, on the whole, commendatory ; but it almost damns with its faint and reluctant praise. Dr. Young says that the theory advanced by Dr. Wells is a consequence so simple and obvious of the principles deduced from the discoveries of Mr. Leslie and other observers, and then generally admitted, that it only required to be distinctly stated and clearly understood, in order to be considered satisfactory. The notice is concluded in the following manner, the allusion in the last sentence being intended for the writer [Dr. Young] himself :—" Had Dr. Wells been as solicitous to attend to the labors of his cotemporaries as he has been very laudably anxious to recur to those of his predecessors, he might have said, not that the experiments of Mr. Prevost *might* be easily accounted for, from the properties he mentions, but that they actually *had* been explained in a similar manner by one of his own countrymen. There are, however, some modern philosophers, who, whether from their own fault, or from that of their hearers and readers, or from both, appear to be perpetually in the predicament of the celebrated prophetess of antiquity, who always told truth, but was seldom understood, and never believed ; and the author of the lectures in question has not unfrequently reminded us of the fruitless vaticinations of the ill-fated Cassandra."*

Dr. Wells replied to Dr. Young, at considerable length, in Thomson's Annals for April, 1815. To the remark of the learned writer, that the theory of Dr. Wells is a simple and *obvious* consequence of principles deduced from the discoveries concerning heat, by Mr. Leslie and other observers, he replies by saying :—" The inquiry of Count Rumford, and the essay of Mr. Leslie, were both published in 1804 ; and in these works are to be found all the new facts relating to heat, which I have taken from others in forming my theory of dew. Whether Count Rumford ever afterwards treated of atmospherical appearances is unknown to me ; but Mr. Leslie published, nine years after his essay, a work on heat and moisture, in which, agreeably to the opinion of Aristotle, the production of dew is attributed to the condensation, by the cold of the night, of watery vapor diffused through a considerable portion of the atmosphere. Now, when the great ingenuity of Mr. Leslie is considered, if the theory of dew, which I have proposed, be an *obvious* consequence of his own discoveries, it would assuredly have occurred to him, in that long space of time, since he has shown that the subject of dew had in the meanwhile occupied his attention."†

Dr. Wells's answers to other portions of the criticism seem to me to be equally fair, manly, and satisfactory. He concludes in the following terms:—" But, I must, on the other hand, be permitted to say that if Dr. Young, forgetting that Newton became a glass-grinder in the service of science, will neglect to employ, for the increase of natural knowledge, the

* Quarterly Review, February, 1814.
† Thomson's Annals, April, 1815.

A few traits of character, picked up from the memoir of his life, will serve in some degree to complete the portrait of Dr. Wells, which I have been endeavoring to sketch. One can hardly help being struck, amongst other things with the *variety* of his occupations, and the *versatility* of his powers. After having crossed the Atlantic to pursue his early studies in Scotland, we find him again in America, a medical student; in a few years, driven by the storm of the revolution, back to Great Britain, and pursuing his medical studies in Edinburgh; doing duty as a surgeon to a Scotch regiment in Holland; imprisoned and finally dismissed the service, for insubordination and rudeness toward his superior officers; preparing his Latin thesis at Leyden; returning from the continent to Scotland and thence to Carolina; looking after the affairs of his family there, and endeavoring to effect a reconciliation between his father and brother; acting at the same time as an officer of volunteers, and a judge advocate in a general court martial of militia officers; engaged as a printer, bookseller and mercantile agent; publishing and conducting a political newspaper in Florida; again captain of a company of volunteers; theatrical manager of a company of young officers, who performed plays for the benefit of the indigent loyal refugees from Carolina and Georgia, achieving great success, he says, in the characters of Lusignan in Zara, and Old Norval in Douglas, but failing in the part of Castalio in the Orphan, and doing still worse in his attempts in comedy; returning from

---

slow and laborious method of observation and experiment, and will frequently exhibit his speculations in a manner, unsuited to the capacities of ordinary men, he ought not to think it strange, that opinions advanced by him on difficult points of philosophy, are not, agreeably to his own remark at the end of the criticism, received as truths beyond doubt, and are often not understood."*

* Thomson's Annals, April, 1815.

Florida to Charleston, to be assailed by mobs, and imprisoned for debt; going back finally to England; engaging in London in public and private practice; enriching the literature of his country with *medical*, *philosophical*, *metaphysical* and *polemical* essays of great ingenuity and remarkable ability; and at last crowning his life and labors with one of the finest achievements of inductive philosophy that the world has ever seen.

It would have been strange and unnatural, if the rough and harsh discipline of his life had failed to leave some of its traces on the habits and temper of his mind—way ward, irritable and impulsive as this naturally was. Dr Garden once attempted to strike him; he eluded the blow, and for three years afterwards, reserved and distant in his manners, he held himself aloof from the society of his teacher and of the young men of the town, and devoted himself seriously and exclusively to his studies.—When acting as surgeon of a regiment in Holland, having received some injury from the colonel of his regiment, he attacked him openly in the street, and challenged him to fight. "I lived," he says, "till I was near eleven years old, close upon the harbor of a large sea-port in America, and by this means associated much with blackguard sailor boys. To this I attribute a practice of swearing, of which I have from the time of being a child, been frequently guilty, when my feelings have been agitated, and even sometimes when no excuse of this kind has existed."* "My temper," he adds in another place, "was naturally irritable, and in small differences which have occurred in society, particularly in my youth, passionate and violent. But, I must, in justice to myself, say, in the first place, I have not shown any considerable instance of this kind,

---

*Works of Dr. Wells, p. 48.

for nearly twenty years; and in the second, that I did never show one, even before that time, in any matter of consequence, or when I had any respect for the person with whom I differed. In confirmation of both these remarks, I shall mention, first, that I have never had the smallest difference with any one of my five most intimate friends; and secondly, that I have borne the grossest insult, when it was unmanly to take immedite notice of it."*

I have already spoken of the conservative character of his political principles. In the course of his memoir, he says, "From the time of the murder of the Princess Lamballe, I foresaw the ruin of all civilized society in France, and dreaded a similar ruin of all civilized society in Europe; I have never, therefore, been able to hear, with the least patience, any serious defence of the conduct of the French; and have always attributed such a defence to incurable folly, self interest, or madness. In all points of domestic politics, I have kept myself free from personal influence, by never seeking the acquaintance of any person of the least influence in the country. By principle I am a constitutional Tory; but my manners, I should think, would lead most persons to regard me a republican."†

Finally, and as we drop the curtain upon this chequered landscape of human life, which for the passing hour we have been contemplating, let us turn our last lingering gaze upon the clouds which envelope it, lighted up with the serene radiance of generous friendship and manly love. With what simple, and beautiful, and touching sincerity does he speak of his friends. "My last declaration," he says, "will relate to the obligations under

---

*Works of Dr. Wells, p. 58.

†Works of Dr. Wells, p. 60.

which I lie to my friends. I have already spoken of my rare good fortune, in having acquired, in the course of my life, five most intimate friends." These friends were David Hume, Mr. William Miller, afterwards Lord Glenlee, Dr. Robertson Barclay, Dr. Matthew Baillie, and Dr. Lister. "All of these," he continues, "are still in being, and from all of them I have received, throughout my illness, the warmest proofs of attachment. Two of them, however, have most especially afforded such proofs, Dr. Lister, and Dr. Baillie, partly from their residing in London, and partly from the nature of their profession. My obligations to Dr. Lister are extreme. During the whole of my disease, he has visited me constantly twice, and sometimes thrice a day; and during each of these visits, he has conducted himself towards me, with fully as much kindness, as if I had been his brother."*

